# Draft genome sequence of a multidrug-resistant *Acinetobacter baumannii* ABMTRAAP01M strain isolated from a dairy cattle farm environment in Bangladesh

**DOI:** 10.1128/mra.00585-26

**Published:** 2026-10-06

**Authors:** Anindita Ash Prome, Md. Monirul Islam, Sadia Afrin Punom, Md. Makshuder Rahman Zim, Amrita Pondit, Saifur Rahman, Md. Bahanur Rahman, Md. Shafiqul Islam, Md. Tanvir Rahman

**Affiliations:** 1 Department of Microbiology and Hygiene, Faculty of Veterinary Science, Bangladesh Agricultural University 54492, Mymensingh, Bangladesh; Portland State University, Portland, Oregon, USA

**Keywords:** whole-genome sequencing, *Acinetobacter baumannii*, MDR, dairy cattle farm, Bangladesh

## Abstract

We report the draft genome sequence of an *Acinetobacter baumannii* ABMTRAAP01M, isolated from the manure of a dairy farm in Mymensingh city, with a total length of 3,431,271 bp, 10 antimicrobial resistance genes, and 29 predicted virulence factor genes.

## ANNOUNCEMENT

*Acinetobacter baumannii* has become a significant global public health concern as it can adapt to challenging environmental conditions such as persisting on abiotic surfaces and potentially develop resistance against several antibiotic classes, may act as a multidrug-resistant (MDR) bacterium, and poses a danger of zoonotic transmission (1, 2).

The draft genome sequence of *Acinetobacter baumannii* ABMTRAAP01M, presented in this paper, was from a dairy cattle farm located in Mymensingh (24.7425°N, 90.4168°E). In July 2025, a manure sample was collected aseptically; after collection, it was transferred to 5 mL of nutrient broth in the lab and incubated aerobically at 37°C overnight (3). The overnight culture was then streaked onto MacConkey agar (HiMedia, India; catalog no. M081-500G) and incubated overnight at 37°C and repeatedly subcultured on MacConkey agar until a pure culture was obtained. Phenotypic identification was done using colony morphology and Gram staining (4). DNA was extracted using the boiling method by suspending a bacterial colony in deionized water, boiling it for 10 min, chilling on ice, centrifuging, and using the resulting supernatant as the template DNA for PCR (5). *bla_OXA51_* primer was used for species identification through PCR (6), then subjected to Sanger sequencing; the GenBank accession number is PX662091 for the isolate. The strain was identified as MDR using the Kirby-Bauer disk diffusion test, which revealed resistance to ceftazidime, meropenem, piperacillin, and tetracycline (7).

After the isolate was grown in Luria-Bertani broth (BD, USA; catalog no. 244620), DNA was extracted using the DNeasy Blood & Tissue Kit (QIAGEN, Germany; catalog no. 69504). The DNA library was then created using the Illumina DNA Prep Reagent Kit (Illumina, USA; catalog no. 20060059). The sequencing was performed using the Illumina NextSeq 2000 platform, producing paired-end reads, and quality checked using FastQC (v0.12.1) (8), and the adapters were trimmed using FastP (v0.23.4) (9). The genome was assembled using SPAdes (v3.15.5) (10); Quast (v5.3.0) (11) was used to evaluate the quality; and the NCBI Prokaryotic Genome Annotation Pipeline (v6.10) was used to annotate the genome (12). ContEst16S was used for contaminant screening (13), while Busco (v5.8.0) via Galaxy Australia was used to evaluate completeness (14). Phylogenetic identification was conducted with Type Strain Genome Server (v401) (15). BacWGSTdb (16) was used for AMR and virulence gene identification, and MLST (v2.22.0) via Galaxy Australia (https://usegalaxy.org.au/) was used to predict the sequence type, indicating possible epidemiological significance (17). The pathogenicity index, antibiotic resistance genes (ARGs), bacteriocin gene clusters, prophages, and metabolic subsystems were predicted using PathogenFinder (v2.0.0) (18), the comprehensive antibiotic resistance database with RGI main (v.6.0.2) (19), BAGEL4 (20), PHASTEST (v1.0.1) (21), and RAST (v2.0) (22), respectively. Unless otherwise noted, default parameters were used for all software.

The genome characteristics of *A. baumannii* ABMTRAAP01M are given in Table 1. The genome comprises 3,431,271 bp with a GC content of 39.05%, identified as sequence type ST515 using seven-gene multilocus sequence typing (*cpn60*, *fusA*, *gltA*, *pyrG*, *recA*, *rplB*, and *rpoB*), and contains 10 ARGs, 29 virulence genes, 302 subsystems, 1 prophage, and 1 bacteriocin. The strain possesses a high predicted pathogenicity score of 0.9849 and is predicted to have human pathogenic capacity.

**TABLE 1:** Characteristics of the assembled genome

Attributes	Values
BioProject accession no.	PRJNA1437284
GenBank accession no.	JBWJBR000000000
BioSample accession no.	SAMN56502588
SRA accession	SRR37640853
Assembled genome size (bp)	3,431,271
Guanine–cytosine (%)	39.05
Sequencing depth (×)	~88
Genome completeness (%)	99.5
Contamination (%)	0.0
N50 value (bp)	54,335
Number of contigs	208
L50	19
Genes (total)	3,375
CDSs (total)	3,326
Genes (coding)	3,141
CDSs (with protein)	3,141
Genes (RNA)	49
rRNAs	1, 2, 1 (5S, 16S, 23S)
Complete rRNAs	1, 1 (5S, 23S)
Partial rRNAs	2 (16S)
tRNAs	41
ncRNAs	4
Pseudo genes (total)	185
CDSs (without protein)	185
Pseudo genes (ambiguous residues)	0 of 185
Pseudo genes (frameshifted)	61 of 185
Pseudo genes (incomplete)	124 of 185
Pseudo genes (internal stop)	31 of 185
Pseudo genes (multiple problems)	27 of 185
Number of ARGs	10
Number of VFGs	29
Genome coverage (×)	105.5
Number of subsystems	302
Pathogenicity score	0.9849
Number of intact prophages	1
CRISPR arrays	2
Paired-end reads	1,312,887
Total reads	2,625,774

## Data Availability

All the data and accession numbers are available in Table 1.
